# COD19 and COD20: An Italian Experience of Active Home Surveillance in COVID-19 Patients

**DOI:** 10.3390/ijerph17186699

**Published:** 2020-09-14

**Authors:** Gian Vincenzo Zuccotti, Simona Bertoli, Andrea Foppiani, Elvira Verduci, Alberto Battezzati

**Affiliations:** 1 Children’s Hospital “Vittore Buzzi”, Azienda Socio Sanitaria Territoriale (ASST) Fatebenefratelli Sacco, 20153 Milan, Italy; gianvincenzo.zuccotti@unimi.it; 2 “L. Sacco” Department of Biomedical and Clinical Sciences, University of Milan, 20157 Milan, Italy; 3 International Center for the Assessment of Nutritional Status (ICANS), Department of Food, Environmental and Nutritional Sciences (DeFENS), University of Milan, 20133 Milan, Italy; simona.bertoli@unimi.it (S.B.); andrea.foppiani@unimi.it (A.F.); alberto.battezzati@unimi.it (A.B.); 4 Italian Auxologic Scientific Institute for Research, Hospitalization and Health Care (IRCCS), 20149 Milan, Italy; 5 Department of Health Sciences, University of Milan, 20142 Milan, Italy

**Keywords:** COD19, COD20, home surveillance, telemedicine service

## Abstract

The severe acute respiratory syndrome coronavirus 2 (SARS-CoV-2) pandemic found Italy unprepared to cope with the large concentrated numbers of patients infected with coronavirus disease 2019 (COVID-19) who often required hospital admission and in many cases intensive care. This pandemic very quickly overwhelmed the Italian Healthcare System. This paper describes the Active Home Surveillance System (Operations Center for Discharged Patients; COD19) and the Home Hospital Care System (COD20) and presents the clinical data collected and the level of user satisfaction with the service. The Operations Center for Discharged Patients (COD19) is an active surveillance service for home-care patients which involves: (1) monitoring critical clinical conditions; (2) recognizing social and health issues; (3) and providing necessary clinical services in the form of a telemedicine service. COD20 is a patient-specialist video consultation service that allows to perform an assessment of clinical conditions and any need to visit; defining the priority of access to specialist outpatient visits in the presence or manageable with the new video consultation model. This service was immediately necessary during the COD19 monitoring. COD19 and COD20 are based on the Amazon Web Services Serverless certified platform. The COD19 and COD20 platform can be intrinsically utilized for future epidemic outbreaks; also those with non-respiratory transmission; and is sufficiently flexible to adapt to natural catastrophes.

## 1. Introduction

The first Italian case of coronavirus disease 2019 (COVID-19) was reported in the Lombardy region on 20 February 2020. The entire epidemic was characterized by a local transmission, with the exception of the first three cases imported from China in late January 2020. The geographical spread of the COVID-19 epidemic appeared immediately highly heterogeneous. In particular, it was very limited in the regions of southern Italy and the islands, on average higher in the regions of central Italy than in the southern regions, and very high in the regions of northern Italy.

As of 18 May 2020 a total of 225,549 COVID-19-positive cases diagnosed by the referral laboratories had been notified to the Integrated National Surveillance System and on 3 April, 2020, Lombardy, the northern region with the highest number of infections, recorded a peak of admissions to the intensive care units (*n* = 1381) [1].

During the pandemic, the province of Milan (the capital city of Lombardy) witnessed a growth in the ratio between cases among its resident population and total cases in Lombardy, the former accounting for approximately 25% of the region’s total cases in the month of May 2020.

This event severely undermined disease management models at the regional level where patients were treated:

(a) In intensive care units (resource rapidly saturated);
(b) In hospital wards reconverted for the purpose;
(c) Increasingly at home, even before clinical recovery, in the event of patients with milder forms of disease, healthy carriers or infected healthcare workers.

Hospital bed occupancy by COVID-19 patients and the closure of most outpatient clinics heavily restricted the healthcare resources available for admissions and the office visits for all the other service users, who were also diverted to home care. Moreover, the problems of disadvantaged patients due to residency in remote areas or limited mobility were exacerbated.

The large number of patients in home care produced a need for appropriate monitoring and management based on the available community care resources—in Italy represented by general practitioners (GPs) and pediatric GPs—to deal with the public health emergency on two levels. The first level, focused on the home-care patient, is able to describe the evolution of monitored parameters, the problems occurring and the final outcomes (mortality, clinical recovery, swab test results, re-hospitalizations, and complications), take action to alert the appropriate provider to clinically critical situations deserving specialist support, and inform the GP. The second level, focused on structuring relations between the players, including the local health agency and the epidemiology service, is able to promptly provide information on the clinical course of home-care patients and the need for new services.

With these premises, a service was set up based on a computer platform that was specially created during the pandemic by the collaboration of the University of Milan, the Health Protection Agency (Agenzia per la Tutela della Salute, Health Protection Agency—ATS) Metropolitan City of Milan and the Local Health and Welfare Agency (Azienda Socio-Sanitaria Territoriale—Local Health Authority, ASST) Fatebenefratelli-Sacco hospitals, and industrial research partners. The aim was to produce a telemedicine service [2,3] allowing the management of home-care patients (virtual hospital) and real-time analysis of the situation to support, using predictive and prescriptive models, the prediction of healthcare system burden and the optimal allocation of resources.

This paper describes the Active Home Surveillance System (COD19) and the Home Hospital Care system (COD20) and presents the clinical data collected and the level of user satisfaction with the service.

## 2. Materials and Methods

### 2.1. COD19

**Figure 1:**
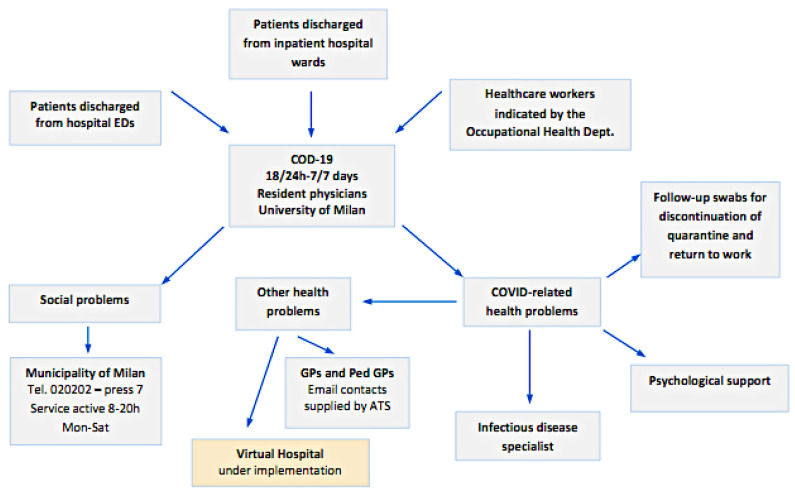
General overview of the Operations Center for Discharged Patients (COD19).

The Operations Center for Discharged Patients (COD19) is an active surveillance service for home-care patients which involves monitoring critical clinical conditions, recognizing social and health issues, and providing necessary clinical services in the form of a telemedicine service (Figure 1). The service is based on a call center that is active 16 h a day, seven days a week.

The following patient classes are subjected to active home surveillance:

- COVID-19-positive patients discharged from the inpatient hospital wards of the ASST hospitals COVID +;
- COVID-19-positive or suspected positive patients (symptomatic but with negative swab) discharged from the Emergency Departments of the ASST hospitals;
- Healthcare COVID-19-positive workers or suspected positive ordered to home quarantine by the Occupational Medicine specialist.

All patients and health professionals falling in these classes were offered the service.

#### 2.1.1. Activation of the Service

Each patient at discharge, or each health professional at the start of his or her sick leave, is given, in addition to the letter recommending home isolation and the indications for fiduciary home isolation, the following materials:

- COD19 kit containing an oxygen saturation meter and a thermometer, as well as individual protection devices;
- Letter communicating the start of COD19 service;
- Instructions for taking the clinical parameters.

#### 2.1.2. Surveillance

It consists of phone calls from resident physicians until the end of the quarantine period to remotely monitor several clinical parameters related to COVID infection. Thanks to the rapid activation of infectious disease consultations, any patient with altered parameters can be referred for prompt admission to the appropriate facility, avoiding inappropriate accesses to the emergency departments.

The first phone contact with the patient takes place within 12 h of receiving the request for activation of the home surveillance. Active home surveillance relies on the use of a protected platform (COD19.it) allowing data collection and processing and connection with the patients, healthcare providers involved, the ATS and the region.

During the active home surveillance, the patient is guaranteed any necessary psychological support and referral to the Social Services of the Municipality of Milan or the municipality where the patient resides.

**Figure 2:**
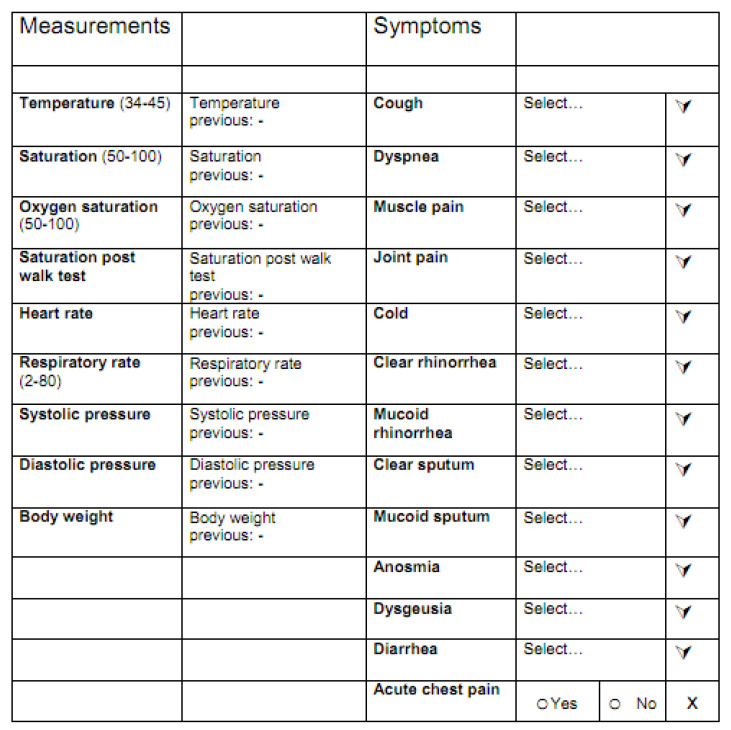
Form for monitoring clinical parameters and symptoms.

For each patient using the service the following clinical data were collected (Figure 2):

- Body temperature;
- Percent oxygen saturation at rest;
- Percent oxygen saturation after a six-minute walk test [4];
- Number of breaths per minute.

Concerning respiratory rate, when accuracy was deemed low, retest were performed with phone assistance of the resident physician performing the monitoring, and when systematic bias was suspected, intra-patient trend more than absolute values were evaluated.

**Figure 3:**
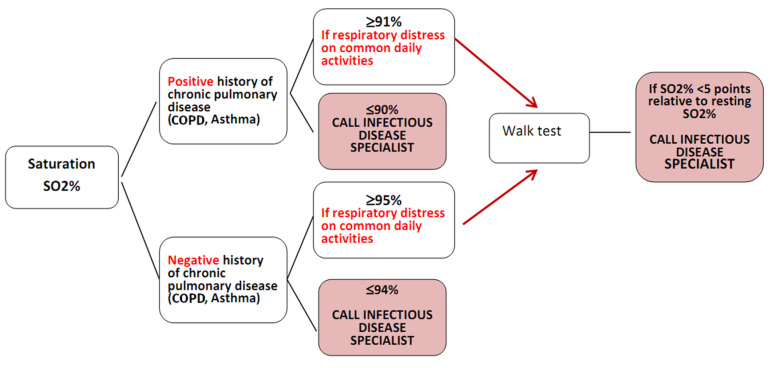
Decision-making algorithm for seeking infectious disease consultation. COPD = chronic obstructive pulmonary disease.

The parameters acquired were interpreted according to the algorithm shown in Figure 3. In the event of altered parameters, the operator emails the reference infectious disease specialist, who instructs on how to proceed with the monitoring or calls the patient directly.

**Figure 4:**
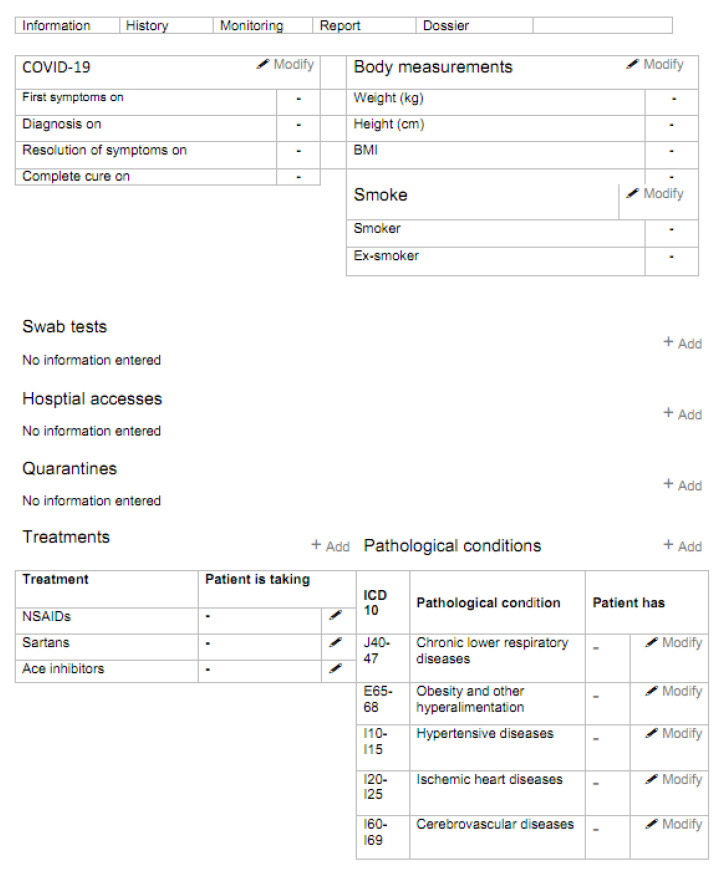
Patient history form.

During the phone call with the patient, the operator also acquires clinical information related to the patient’s history of COVID-19 and underlying comorbidities (the data collection form is shown in Figure 4). Within 12 h, the patient’s GP is notified by email that the patient is under the care of the Active Home Surveillance Service.

**Figure 5:**
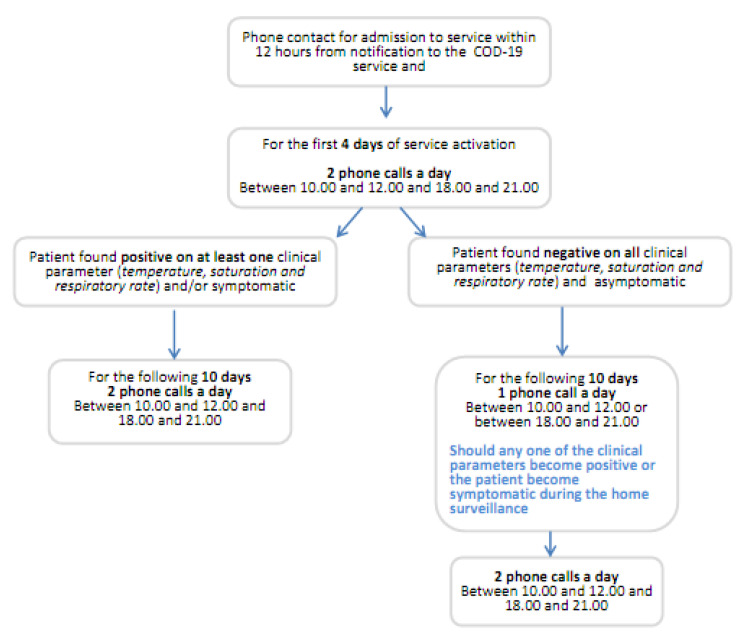
Algorithm for the management of active home surveillance over the 14 days.

Should all the patient’s clinical parameters prove negative on four consecutive days, active home surveillance is reduced to only one phone call per day, as shown in Figure 5.

#### 2.1.3. Management of Swab Tests

The disappearance of symptoms does not correspond to microbiological cure and the patient may still be contagious. For this reason, while at home they will have to observe a period of fiduciary quarantine, remaining in isolation and avoiding contact with other people for at least 14 days after disappearance of the symptoms. During home surveillance, the recording of values of body temperature >37.5 °C, or respiratory rate >22 breaths per minute, oxygen saturation ≤94% or a reduction in oxygen saturation of over five points following the walk test entails recalculation of the days defining clinical recovery starting from the first day on which all the above parameters are found to be negative. At the end of the 14 days of isolation, the patient undergoes two follow-up swab tests, to be taken 48 h apart, in accordance with the instructions provided by the ASST service.

**Figure 6:**
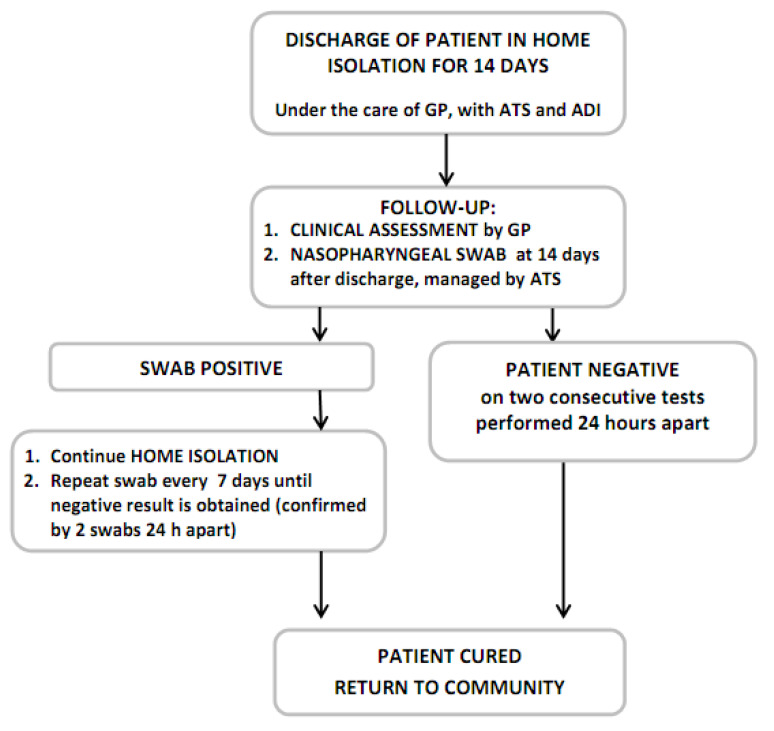
Flowchart showing the management of swab tests.

Should the two swabs both prove to be negative, this means the infection has completely resolved, the virus is absent and the quarantine period can end. If one or both swabs are positive, they have to be repeated after one week and the home isolation is continued (Figure 6).

#### 2.1.4. Satisfaction Questionnaire

**Figure 7:**
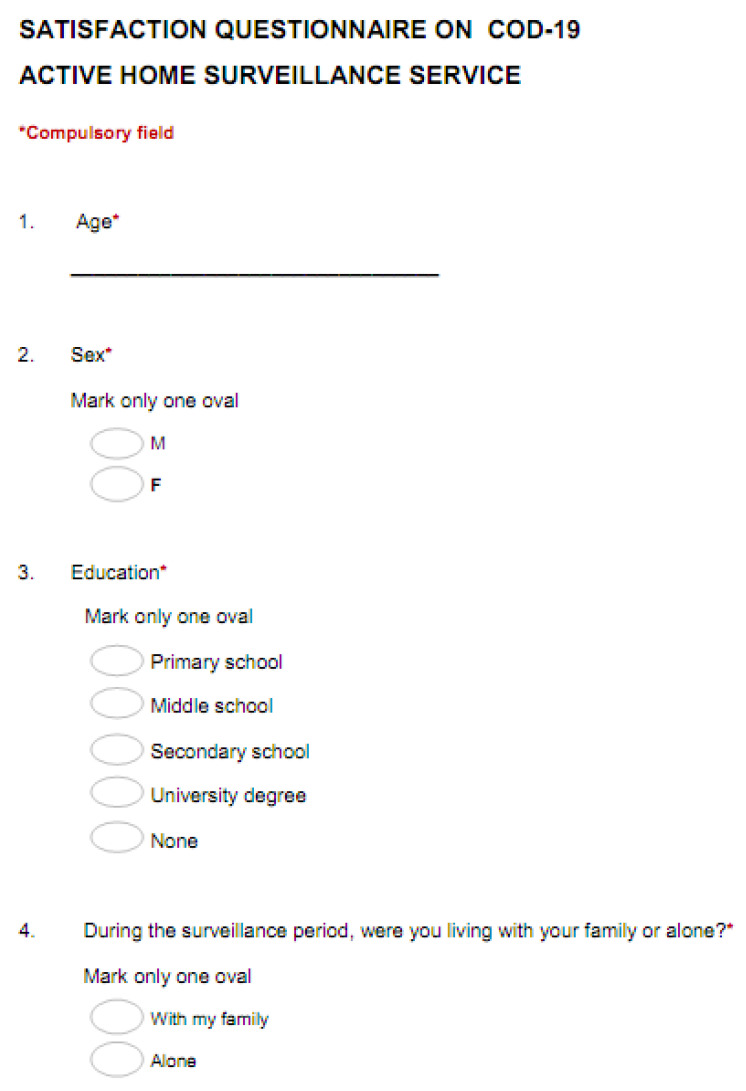
Questionnaire on satisfaction with the service.

After the 14 days of active home surveillance, patients receive a satisfaction questionnaire (Figure 7) investigating their relationship with the care provided, ease of measurement of the clinical parameters, quality of the services used and overall satisfaction with the service, by expressing a categorical judgement (not at all, a little, somewhat, very, extremely).

### 2.2. COD20

COD20 is a patient-specialist video consultation service [5,6] that allows you to perform an assessment of clinical conditions and any need to visit, defining the priority of access to specialist outpatient visits in the presence or manageable with the new video consultation model. This service was immediately necessary during the COD19 monitoring following the detection of comorbidity in the monitored patients, but it also represents an important management opportunity during the gradual mitigation of the lockdown in Italy.

The COD20 service provides for an automation and simplification of the visit booking process, which takes place through integration with the regional single booking center (centro unico prenotazioni—CUP). The visit is provided through video-consultation through web-based technologies, which do not require any preparation of the patient’s personal devices. Following the video consultation, the report is archived in the dossier for future reporting and for possible publication in the electronic health record.

The dossier function also simplifies files exchange between patients and specialists, avoiding the need of third-party software, giving full data governance to the patient.

The video is also fully integrated, and this not only useful for security, but also to keep track of quantitative and qualitative indicators.

### 2.3. Digital Platform

COD19 and COD20 are based on the Amazon Web Services (AWS) Serverless certified platform. It is multitenant, many hospitals can join with their own data, and use the same engine. The Lambda serverless infrastructure means service continuity, continuous feature updating, and dimension scalability. The application programming interface (API) gateway is used for microservices to enable easy and secure interoperability, within internal software blocks, and external data sources. That means that every function is an independent block, with no dependencies between them. For example, a hospital can use its login system, and use the rest of the functions. Another operating unit can use its own messaging system and use the rest of the platform. The database used was Aurora—for speed and scalability. Business intelligence (BI) is based on AWS Quicksight. Security is centrally monitored and managed natively by AWS infrastructure. When evaluating costs, take into consideration that pricing is over exact usage metrics, and includes security, continuity, and future proof infrastructure updating. Furthermore, the speed of development workflows frees highly costly developer resources. Agile/Scrum project management is based on Trello, and Telecom on FreePBX.

## 3. Results

Over 58 days of activity, the service took charge of 1097 patients and conducted a total of 27,195 calls. For those patients who have already completed the monitoring, the median follow-up was 20 days. A total of 38% of patients were referred to the service following discharge from a hospital unit, 40% following discharge from an emergency department and 22% were referred by the occupational medicine service. A total of 58% of patients were women and the median age was 48 years.

Although, in most cases, the disease course had a positive outcome, 52 patients under surveillance were admitted to hospital following detection of pathological parameters during the surveillance period (all patients were eventually discharged as none of the readmission resulted in exitus). The actions undertaken as a result of the phone calls include 424 contacts with the infectious disease specialist for assessment and discussion of parameters and symptoms related to COVID-19 and 72 patients referred for psychological support based on the results of the depression and anxiety screening tests.

As an approximate measure of household transmission during home isolation, we also report results from patients sharing the same household. Among our patients, 76 shared households were recorded for a total of 163 patients. While most of these patients were referred to the service at the same time, 28% were referred during the monitoring of the first household members. While it cannot be known for sure if transmission happened during home isolation or before, 4% of patients were referred after 14 days from the first phone call of the first household member, increasing the probability that transmission happened during home isolation.

At the time of admission to the service, the patients’ clinical parameters were distributed among the three patient groups as shown in Table 1. During monitoring, 39% of patients had alteration of at least one parameter among temperature, oxygen saturation and respiratory rate. In particular, 4% had at least one episode of temperature >37.5 °C, 13% a saturation lower than 90 or 95% (varying depending on underlying condition) and 31% a respiratory rate >22 breaths per minute.

**Figure 8:**
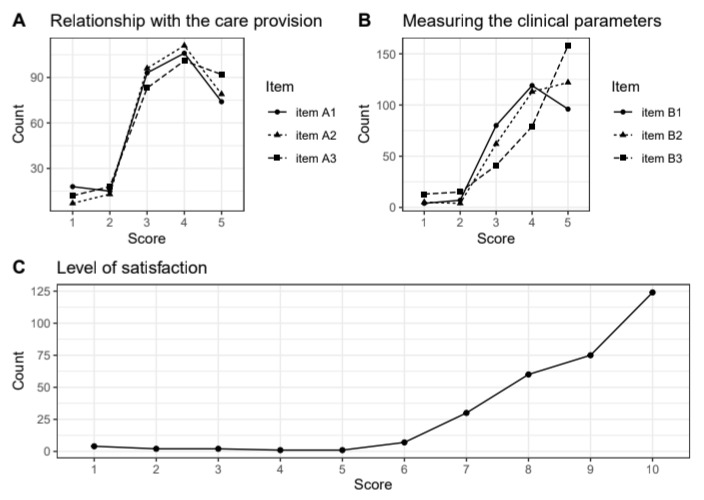
Summary of responses on satisfaction with the service. A high score indicates a positive judgement. Panel (**A**) summarises scores related to completeness of initial information provided by COD19 operators (Item A1), clarity of information received (Item A2), and clarity of instructions on measuring equipment (Item A3). Panel (**B**) summarises scores related to the ease of daily measurements (Item B1), ease of measuring parameters by themselves (Item B2), and preference of a phone call vs. monitoring by applications or digital devices. Panel (**C**) summarises the overall satisfaction with the service.

Overall, the service was well accepted by the patients. Of the 306 patients responding to the satisfaction questionnaire, only five gave at least one negative rating of the relationship with the care provision, ease of measuring the clinical parameters or level of general satisfaction with the service (Figure 8). Negative ratings came from asymptomatic patients, and on this feedback, we adjusted the follow-up frequency of asymptomatic patients to once a day after four days without symptoms.

## 4. Conclusions

The severe acute respiratory syndrome coronavirus 2 (SARS-CoV-2) pandemic hit Italy hard and suddenly, and found the country unprepared to cope with large, concentrated numbers of patients infected with COVID-19 who often required hospital admission and in many cases intensive care. This pandemic not only overwhelmed the Italian Healthcare System, it also generated concern and isolation among the population. Hospitals became rapidly saturated, partly because the community care services proved unable to contrast the phenomenon in a prompt and timely manner. The result was a major separation between hospital care and community care services, with isolation at home of COVID+ patients as well as the rest of the population, in an attempt to contain the spread of the infection. Hence, the need to set up a facility able to bridge the gap and facilitate the flow of patients from the hospital (intended as intensive care units and wards) to the community (intended as GPs and pediatric GPs) and vice versa. A facility able to accompany patients discharged from hospital to community care and those in the community requiring rapid access to hospital, while monitoring patients with COVID-related symptoms in isolation at home.

This intervention model follows other notable applications of telemedicine in managing pandemics [7] and is nested in broader conceptual frameworks proposed to fight the COVID-19 pandemic [8], developing specific strategies on two of the main points of the framework: remote patient monitoring and tele-expertise with specialists. A natural next step for this model would be the monitoring of asymptomatic individuals in active outbreaks or case contacts, to promptly triage cases with suspicious symptoms. This would allow monitors to readily dispatch medical assistance or start stricter monitoring, based on severity criteria. Similar models were already applied in the Ebola virus disease outbreak [9,10].

This model, as well as being crucial in managing the COVID emergency, provides a template for remote clinical intervention also applicable to future emergencies [11]. The virtual hospital, and its ability to integrate with the regional health system (access to healthcare databases and their integration with the surveillance data, reporting of remotely provided health services), will play a central role in phase two of the epidemic as in-person office visits will have to be reserved for those few cases or single services for which virtual visits are unsuited or ineffective. Indeed, clinics will not return to pre-pandemic capacity as the social distancing rule prohibits the presence of several persons in a confined area, and the time patients spend in the clinic will have to be limited. It will, therefore, be mandatory to carry out, through the virtual hospital, remote initial assessments, which will generate and schedule the subsequent in-person visits, as happened in Catalonia, Spain during the COVID-19 pandemic [12].

This model was indeed well accepted during the pandemic crisis by the regional health system and found low implementation barriers, but even in the future normal clinical practice, the virtual hospital may represent an increasingly utilized resource, characterized by relatively low operational costs and general patient acceptance. The flexibility to use low level, but widely available technology, such as phone calls to more advanced implementation permits high applicability in other part of Italy or the rest of the world. The high patient acceptance of active monitoring also contrast with low adoption of app-based passive monitoring recorded during this pandemic.

This model will have an impact on the ability to monitor in real time citizens’ state of health and needs in those settings, such as the home setting, that normally escape close control. It will also have an impact on healthcare management by allowing resource optimization and allocation, an impact on institutional decisions through the development of operational research models and algorithms and an undoubted scientific impact.

COD19 is still active and will be extended in the future, based on the experience collected in these months and other telemedicine implementations [13], to generate predictive and prescriptive models to better manage future patients. The COD19 and COD20 platform can be intrinsically utilized for future epidemic outbreaks, also those with non-respiratory transmission, and is sufficiently flexible to adapt to natural catastrophes. Thanks to its multitenant features, it can serve several healthcare facilities, which share the model and engine but can personalize functions, with near-instant speed. Another innovative component is the intention to adopt this service as a new layer of clinical healthcare service processes, thus perfectly integrating it into the hospital information systems and avoiding its isolation as a separate entity.

## Figures and Tables

**Table 1:** Parameters on admission to the active home surveillance service.

Referrer	Parameter	Mean ± SD	Quartiles
Discharged from hospital ward	Respiratory rate	18.9 ± 5	16/19/22
	Saturation	97.2 ± 1.3	97/97/98
	Saturation after 6—min walk test	96.6 ± 1.7	96/97/98
	Temperature	36.1 ± 0.5	35.8/36/36.4
Discharged from Emergency Dept.	Respiratory rate	18.9 ± 6.5	16/18/22
	Saturation	96.8 ± 8.8	97/98/98
	Saturation after 6—min walk test	94.4 ± 14.1	96/97/98
	Temperature	36.3 ± 0.6	36/36.3/36.7
Occupational Medicine	Respiratory rate	18 ± 4.1	16/18/20
	Saturation	97.8 ± 0.9	97/98/98
	Saturation after 6—min walk test	95.5 ± 9.1	95.8/97.5/98.2
	Temperature	36.4 ± 0.5	36/36.4/36.8

## References

[1] Higher Institute of Health, Italy COVID-19 Integrated Surveillance: Key National Data. https://www.epicentro.iss.it/en/coronavirus/sars-cov-2-integrated-surveillance-data.

[2] Tuckson R.V., Edmunds M., Hodgkins M.L. (2017). Telehealth. N. Engl. J. Med..

[3] Hollander J.E., Carr B.G. (2020). Virtually Perfect? Telemedicine for Covid-19. N. Engl. J. Med..

[4] Enright P.L. (2003). The six-minute walk test. Respir. Care.

[5] Calton B., Abedini N., Fratkin M. (2020). Telemedicine in the Time of Coronavirus. J. Pain Symptom Manag..

[6] Smith A.C., Thomas E.E., Snoswell C.L., Haydon H.M., Mehrotra A., Clemensen J., Caffery L.J. (2020). Telehealth for global emergencies: Implications for coronavirus disease 2019 (COVID-19). J. Telemed. Telecare.

[7] Ohannessian R. (2015). Telemedicine: Potential applications in epidemic situations. Eur. Res. Telemed./La Rech. Eur. en Télémédecine.

[8] Wu C., Liu Y., Ohannessian R., Duong T.A., Odone A. (2020). Global Telemedicine Implementation and Integration Within Health Systems to Fight the COVID-19 Pandemic: A Call to Action. JMIR Public Health Surveill..

[9] World Health Organization Statement on the 1st Meeting of the IHR Emergency Committee on the 2014 Ebola Outbreak in West Africa, 2014. https://www.who.int/mediacentre/news/statements/2014/ebola-20140808/en/.

[10] World Health Organisation Busting the Myths about Ebola Is Crucial to Stop the Transmission of the Disease in Guinea, 2014. https://www.who.int/features/2014/ebola-myths/en/.

[11] Alwashmi M.F. (2020). The Use of Digital Health in the Detection and Management of COVID-19. Int. J. Environ. Res. Public Health.

[12] Sust P.P., Solans O., Fajardo J.C., Peralta M.M., Rodenas P., Gabaldà J., Eroles L.G., Comella A.C., Munoz C.V., Ribes J.S. (2020). Turning the crisis into an opportunity: Digital health strategies deployed during the COVID-19 outbreak. JMIR Public Health Surveill..

[13] Tolone S., Gambardella C., Brusciano L., Del Genio G., Lucido F.S., Docimo L. (2020). Telephonic triage before surgical ward admission and telemedicine during COVID-19 outbreak in Italy. Effective and easy procedures to reduce in-hospital positivity. Int. J. Surg..

